# Use of Integra® as a Temporizing Measure Before Definitive Flap Surgery in Mangled Extremities Requiring Revascularization

**DOI:** 10.7759/cureus.16053

**Published:** 2021-06-30

**Authors:** Uzair Qazi, Laxminarayan Bhandari

**Affiliations:** 1 Hand Surgery, Christine M Kleinert Institute for Hand and Microsurgery, Louisville, USA

**Keywords:** vein graft, emergency revascularization, hand injuries, hand and microsurgery, limb replantation, crush injuries, limb-salvage

## Abstract

Complex palm defects with devascularized digits often require vein grafts. This may need to be followed by a free or pedicled flap to resurface the tissue lost and also cover the vein grafts and the anastomosis. However, if the anastomosis fails, or there is marginal wound necrosis, the existing flap would be redundant and a different flap would be needed for coverage.

In such situations, a novel approach would be to temporarily cover the anastomotic site with Integra® (Integra LifeScience Corporation, Plainsboro, NJ), until the digit and skin margin vascularity stabilize. This technique can be used to buy time and potentially lessen the need for pedicled or free flap coverage in the first instance. This should be taken into consideration as a viable alternative during revascularization in such cases.

In this case report, we present two cases in which we used Integra® as a temporary cover over exposed vein grafts until vascularization was established and a more definitive flap coverage was done. We were able to place the flap directly over the Integra®. This allowed time to determine additional necrosis of the wound margins and the final size of the defect that required coverage. Other advantages of this technique are that Integra® is readily available, there is no donor site morbidity, and the silicone layer which is not directly in contact with the vein graft can be easily peeled off without any traction. A flap or skin graft can be done directly over the collagen layer of the Integra®. Also, having a collagen layer integrated over the vein graft can potentially obviate the need for a flap.

## Introduction

Reconstruction of complex hand injuries that involve devascularization of digits and extensive soft tissue defects requires careful planning. Management of such injuries includes, but is not limited to, skeletal stabilization, repair of tendon and nerves, revascularization followed by soft tissue coverage. Vein grafts are often required to revascularize the digits. These vein grafts need soft tissue coverage preferably using a flap [1].

The survival of digits is often not certain in severely mangled extremities and affected digits are lost. In addition, the native tissue in the zone of injury takes time to demarcate and it is difficult to determine its viability during the initial surgery [2]. If flap coverage is performed primarily, it might result in inadequate coverage due to possible wound necrosis, expanding the existing defect size. Also, if the fingers do not survive, the patient may need an additional flap in anticipation of a toe-to-hand transfer.

Hence, a temporizing measure before definitive coverage would be very useful in the reconstructive algorithm. Such a procedure would allow time to establish revascularization and demarcate the zone of injury [3]. We describe a temporizing technique using Integra® (Integra LifeScience Corporation, Plainsboro, NJ) and our experience in two cases of devascularized complex hand injuries.

## Case presentation

Case 1

**Figure 1:**
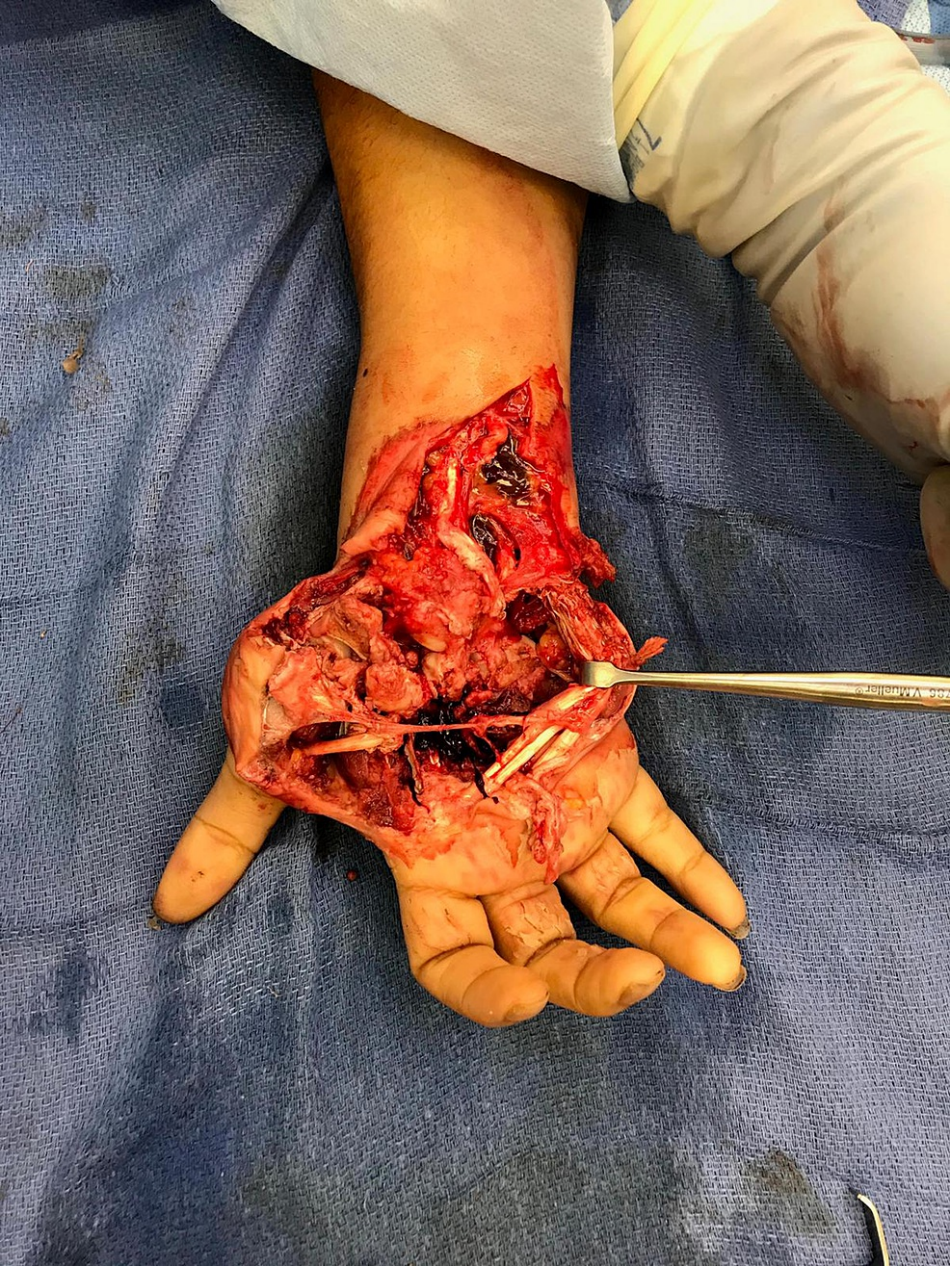
Hand defect showing the extent of the injury and devascularization.

**Figure 2:**
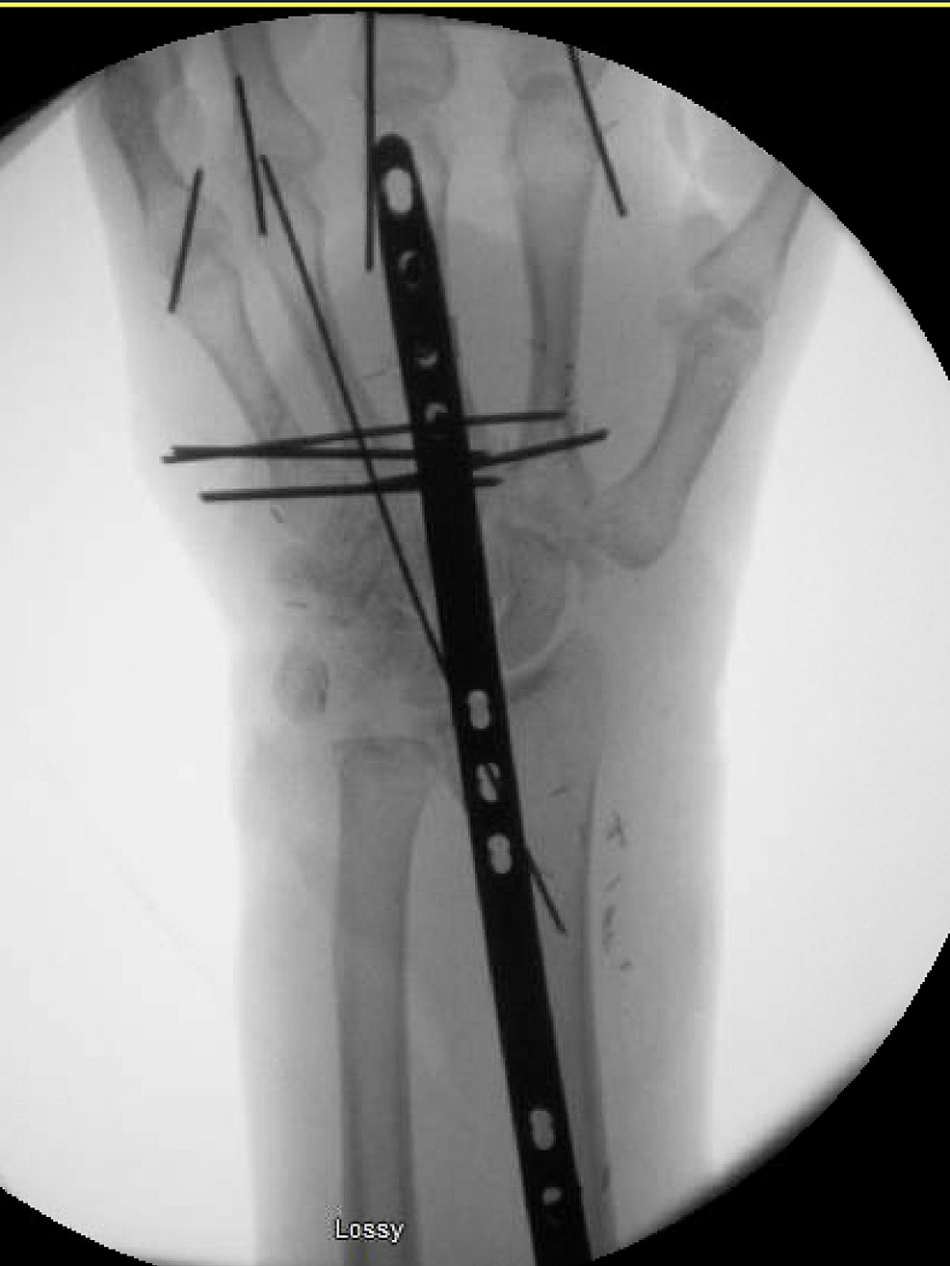
X-ray showing stabilization of the metacarpal fractures with K-wires and a spanning wrist plate placed to stabilize the wrist.

**Figure 3:**
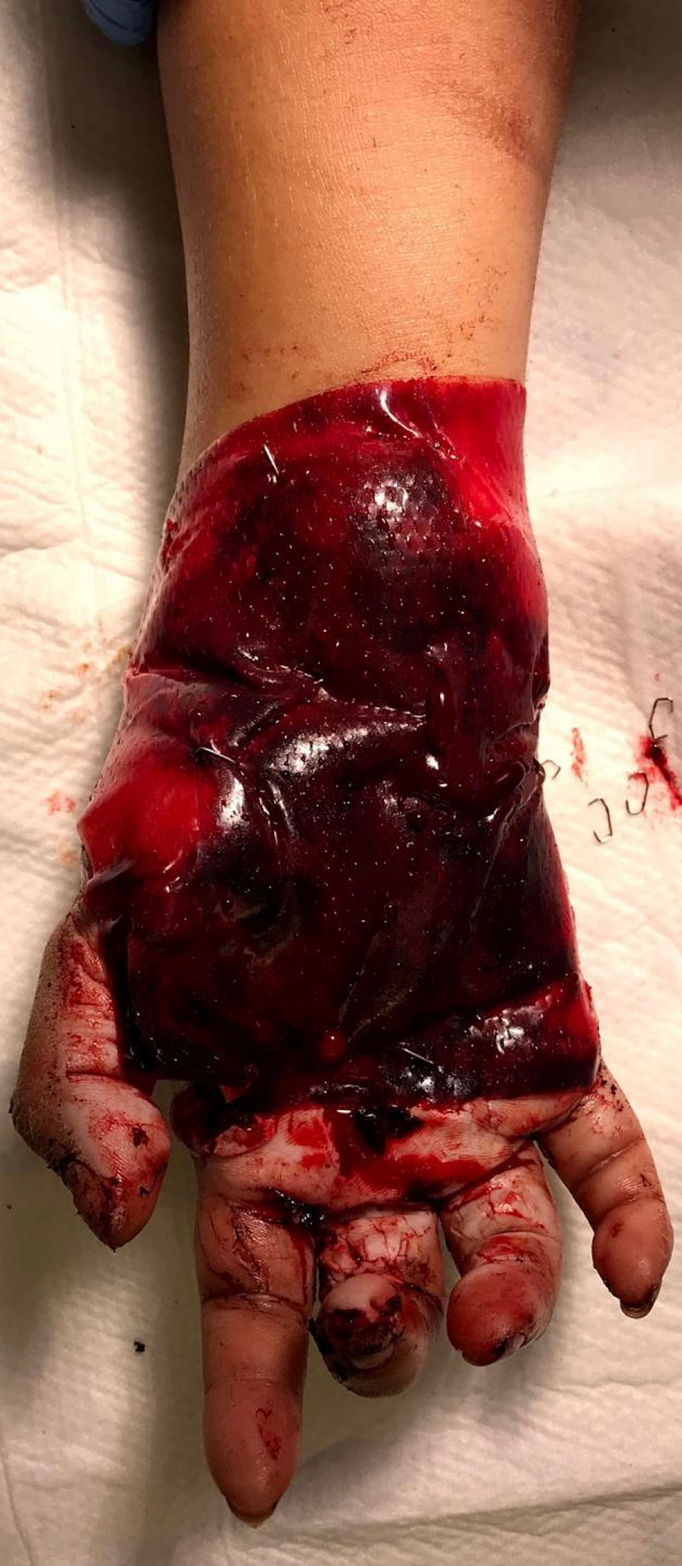
Application of Integra® as a temporary cover over the vein graft and soft tissue defect.

A 32-year-old woman’s right hand was severely crushed (mangled extremity severity score {MESS} 9) in a tortilla maker. The patient had a loss of distal carpal row and also sustained multiple metacarpal fractures with segmental loss of ulnar and median nerves, flexor tendons, superficial palmar arch, and common digital arteries. The hand was devascularized in addition to a palmar skin defect measuring approximately 8x10 cm (Figure 1). After stabilizing the metacarpal fractures with K-wires, a spanning wrist plate was placed to stabilize the wrist (Figure 2). Nerve allografts were used to bridge the segmental loss of median and ulnar nerves. A dorsal vein from the right foot was used as an interposition vein graft to reconstruct the superficial palmar arch. All the common digital and proper digital arteries were anastomosed end to side to the vein graft in order to revascularize the digits. We applied Integra® as a temporary cover over the vein graft and soft tissue defect (Figure 3). 

**Figure 4:**
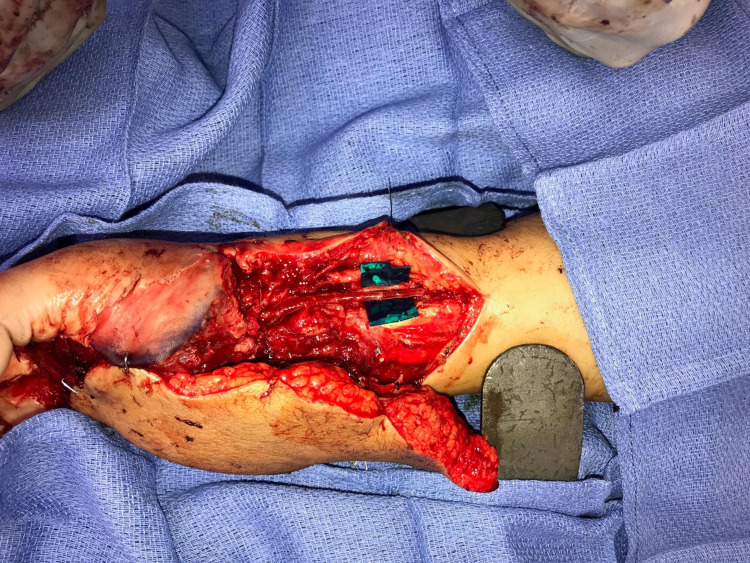
Free lateral arm flap to cover the defect anastomosing the flap artery end to side with the radial artery.

On postoperative day three, all her digits were viable; however, there was further palmar wound necrosis resulting in a defect larger than appreciated initially. A free lateral arm flap was done to cover the defect anastomosing the flap artery end to side with the radial artery (Figure 4). We were also able to place the flap directly over the Integra without removing it thereby, protecting our vein graft and anastomosis.

**Figure 5:**
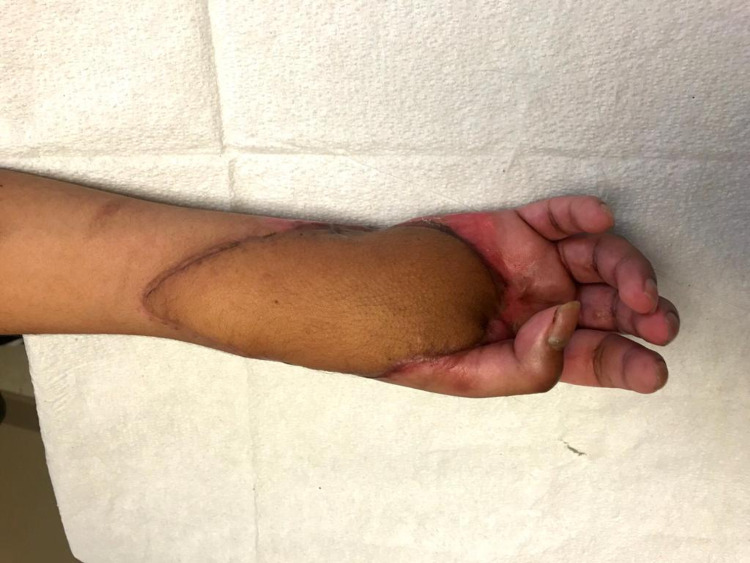
A follow-up picture showing a well-healed and contoured flap.

The patient recovered uneventfully and subsequently underwent secondary procedures for plate removal and flap thinning and flexor tendon reconstruction (Figure 5).

Case 2

A 22-year-old woman had a crush injury (MESS 9) to the right hand with a press machine. There was a complete loss of the index and long fingers. The small and ring fingers had a mangling injury with metacarpal and proximal phalangeal. These digits had a loss of vascularity as well.

She also had volar and dorsal skin defects and loss of the palmar arch. After skeletal stabilization with K-wires, a vein graft was harvested from the dorsum of the right foot. One end was anastomosed to the ulnar artery while the other end was ligated. The proper digital artery to the small finger and second and third common digital arteries were anastomosed end to side with the graft. There was extensive soft tissue loss on the dorsum of the hand and the ring finger and soft tissue loss on the palmar aspect of the hand. Similar to the first case, in order to ensure revascularization of fingers and stabilization of the trauma zone, we used Integra® as a temporary cover.

An increase in defect size due to marginal necrosis was noticed in this case as well. On the fifth postoperative day, after removing the silicon layer of the Integra®, a groin flap was done to cover the dorsum of the hand and a split-thickness skin graft was placed over the dorsum of the ring finger and the hypothenar area.

At follow-up after two weeks, there was a loss of skin graft due to infection, but the groin flap and finger vascularity remained unaffected. She was taken for surgery again at three weeks and the groin flap was divided, the defect on the palm was skin grafted again and the dorsum of the ring finger which had a skin graft loss was covered with a transposition flap elevated from the divided groin flap.

Expected outcome

Both of our patients’ hands survived, and their vein grafts and the anastomotic sites remained patent until we were able to achieve definitive flap coverage. This temporizing measure provided adequate time for any ischemic region to demarcate itself and digit revascularization to be confirmed. It also allowed patients to be stabilized and adequately counseled about their injury and treatment options.

The skin graft loss in our second case could be due to a combination of infection and premature application over Integra®, without adequate time for integration. However, the vein graft under the collagen layer remained patent and allowed for skin grafting later. We were able to graft it again at three weeks during groin flap division.

## Discussion

While primary flap coverage of complex hand injuries has many advantages, such as early coverage, and early initiation of rehabilitation, two main issues need due deliberation. Firstly, the zone of trauma may not demarcate well at the time of initial injury. A flap based on the initial defect size may turn out to be inadequate later. As we noticed in both our cases, the final defect size was larger than the initial defect post debridement. This is especially true if considering a distant pedicled flap, such as a groin flap, where the revascularization of the flap would depend on the wound margins being healthy. Secondly, the treatment plan in mangled hand injuries would be altered based on the viability of the fingers. If the fingers failed to survive, an appropriate flap would be designed keeping in mind the future need for a toe transfer. Such a flap would be of a different dimension and orientation compared to a flap used for coverage of the defect [4]. A temporizing coverage in such a situation would allow for adequate time to establish vascularity and the zone of trauma to stabilize.

The temporizing options available are negative pressure wound therapy (NPWT), skin graft, or Integra application. Direct application of NPWT sponges over the vein graft is not preferable. The compression of the vein graft and the risk of pulling the vein graft or disrupting the anastomotic sites during dressing change are significant risks. Another temporizing option is skin grafting before definitive flap cover. The graft would need to be removed or de-epithelized before flap coverage and this confers a risk on the underlying vein graft. The donor site morbidity is an additional factor to consider.

In the current study, we describe a technique where we used Integra® as a temporary coverage over vein grafts used for revascularizing digits. The advantages of this technique are that Integra® is readily available, there is no donor site morbidity, the silicone layer which is not directly in contact with the vein graft is easily peeled off without any traction [5]. A flap or skin graft can be done directly over the collagen layer of the Integra®. Also, having a collagen layer integrated over the vein graft can potentially obviate the need for a flap [6].

Advantages of this technique are that it gives us more time to assess and evaluate the viability of the revascularized digits and adjacent soft tissues, it allows time to plan the appropriate coverage options, it temporizes and reduces the time of the initial surgery which is very important in polytrauma patients, there is no added donor site morbidity and NPWT can be applied over the Integra® in selected cases [7]. This is also an easily reproducible technique.

There are a few limitations as well which include the additional cost of Integra®, skin grafting cannot be performed immediately over the Integra® [8]. 

## Conclusions

Integra® is useful as a temporary cover over vein grafts following revascularization in severely mangled extremities. It provides critical time to assess digit survival as well as the viability of tissue within the trauma zone. This gives the surgeon time to select the most suitable option for definitive reconstruction. Compared to negative pressure wound therapy or a skin graft, Integra® can be directly applied over a vein graft. During the definitive surgery, the silicon layer can be removed, and the collagen layer can be left in place. Overall, this could prove to be a useful addition to the armamentarium of temporizing measures in such situations. However, more studies with larger cohorts and longer follow-ups are required to assess long-term outcomes.
